# TERRITORY AS A SOCIAL CONSTRUCTION

**DOI:** 10.1590/S0104-59702024000100024en

**Published:** 2024-06-07

**Authors:** Renato da Gama-Rosa Costa, Inês El-Jaick Andrade

**Affiliations:** i Departamento de Patrimônio Histórico/Casa de Oswaldo Cruz/Fiocruz. Rio de Janeiro – RJ – Brazil; ii Departamento de Patrimônio Histórico/Casa de Oswaldo Cruz/Fiocruz. Rio de Janeiro – RJ – Brazil

Over the past four decades, the category of “cultural heritage” has expanded to the point that it spans a wide range of objects, materials, spaces, social practices, and knowledge that are identified, celebrated, and/or contested as heritage by one or more social groups (Gonçalves, 2015). This expansion occurred alongside reassessments that address the aspect of interaction between materiality and immateriality, matrixes of values such as attributions, social participation in the process of creating heritage and managing heritage resources, and more recently, various dimensions of the effects of the colonial process.

Brazil’s 1988 Constitution, which notably included social participation, established that the government (in cooperation with the community) is responsible for protecting manifestations of popular and indigenous cultures, as well as those of African descendants and the other groups that comprise Brazilian society. The country’s cultural heritage is defined as the material and immaterial assets that refer to the memory, identify, and activity of these groups.

While ethnic, social, and religious minorities have made significant advances in the struggle for representation over the past decade, especially in recognition of their immaterial cultural references, their territorial identities still remain detached. This is a contradiction, since heritage is linked to territory and memory, which both serve as vectors of identity (Hartog, 2014); the collective memory of a social group requires a territorial reference. Territory is consequently the record of the tensions, successes, and failures in the history of a society, and needs to be understood within an integrating perspective (Haesbaert, 2004).

In this scenario, territory as a social construction (a place) emerges as a locus of resistance and mobilization. Talking about cultural heritage in the territory of Manguinhos means bringing the historical subjects and individuals from this space to the center of the discussion, recovering significant processes, experiences, and events that were erased from the dominant narrative, as well as recognizing and valuing the active role they played and their knowledge. Historically, interventions on the constructed and social environment in favela communities tended to not be planned, implemented, or evaluated with regard to the processes that are critical in local determination of health, even though they offer the potential to reverse health inequity. Additionally, the impacts that planned decisions and their implementation will have on the territory and on the population’s health and quality of life are rarely considered, and the population as a rule is not included in the decision-making process or social scrutiny of the activities carried out within the context of their lives.

In order to foster this debate with regard to recognition of territorial identities and territorialities and their influences on health processes, we proposed the publication of this supplement in 2023, the year marking the twentieth anniversary of the UNESCO Convention for the Safeguarding of Intangible Cultural Heritage (32nd session), 40 years since the establishment of UNESCO’s Living Human Treasures program, and the thirty-fifth anniversary of the Brazilian Federal Constitution.

The supplement covers different forms of production in the urban space of Manguinhos, the headquarters of the Fundação Oswaldo Cruz (Fiocruz), through articles that use different focuses and scales to address influences and landmarks in the region that reflect the spatial and cultural development of this space in some way. It consequently provides a space for interdisciplinary reflection on the relationship between territory and local identity, including tangible and intangible aspects, especially related to health and cultural heritage.

Within this context, the supplement is divided into three different sections and contains a total of six articles: four in “Analysis,” one in “Images,” and another in “Interview.”

The opening article in the supplement, “A history under construction: Manguinhos-Maré in the present time,” by Renato da Gama-Rosa Costa, Renata Soares Costa, and Matheus Gonçalves, reviews the first two decades of the twenty-first century and shows how this era was representative of the institution’s history, confirming the leading role played by Fiocruz within the national health scenario. This institution envisioned advances in research and in disease diagnostics, together with expansion of its infrastructure network (the inheritance of an identity forged since the start of the twentieth century) with regional and international coverage, at the same time that financial and sanitary crises erupted. The article describes the spatial transformations in the territories occupied by Fiocruz, especially the Fiocruz Manguinhos-Maré campus, from 2000 onward. The transformations in the use of these territories are linked to Fiocruz’s institutional policy within the national context, and the social demands that impacted the institution (large-scale urban transformations and pandemics) are emphasized. This study uses the assumptions of present-day history to investigate the changes that took place on the Manguinhos-Maré Campus during this period and in the discourse of the institutions involved in managing and planning these changes. To do so, it combines an analysis of the institutional reports and digital mapping of the institution, as well as aerial images obtained from the municipality to assist in investigating the physical changes that took place on the campus.

In “Urban health, the right to the city, and the communities of Manguinhos in Rio de Janeiro,” Luis Carlos Soares Madeira Domingues and Rosângela Lunardelli Cavallazzi study the historical and contemporary dynamics of urbanization in Latin America. They start from the understanding that this dynamic produces and reproduces inequalities, emphasizing the urban crisis that currently affects us and penalizes most of the population by concentrating health inequalities in the areas of greatest urban poverty, namely favela and peripheral communities. Brazilian cities in particular spatially reflect the severe historical and structural imbalances of our society. One of the challenges especially related to setbacks in public policies and social rights is reflection which is also associated with facing the situations that emerge in supporting families in vulnerable territories with medium- and long-term proposals that can transform the processes of social determination associated with the limiting habitational and urbanistic factors that affect the health and quality of life of this population, as well as the conditions involved in facing these and future health and climatic emergencies. The objective of this work is to contribute to this reflection, considering the theoretical methodology of urban fieldwork and associating a critical approach to epidemiology and urban theory. This approach is intended to surpass a purely functionalist perspective between factors and the health/disease process while also considering the importance of the historical and social dynamics and the complexity of urbanistic analysis of the built environment and its social production and reproduction. This work uses the favela communities of the Manguinhos Complex (which were the subject of the Favela Urbanization Growth Acceleration Program, or PAC) as a reference case.

In “Social participation and territory: possible dialogues for the sustainable management of cultural heritage,” Marcos José de Araújo Pinheiro and Roberta dos Santos de Almeida work from an anthropological understanding of the concept of “cultural heritage” as a set of things, relationships, and meanings attributed by different groups and individuals in society and bring together a perspective of integrated conservation and sustainability in such a way that reaffirms the importance of social participation in the processes of constructing, preserving, appropriating, valuing, caring, and transmitting heritage. Their topic of study is the Manguinhos Historical Architectural Complex (NAHM), a set of buildings of historical and cultural interest located within Fiocruz’s Manguinhos campus, as well as its 2014 Requalification Plan to expand the range of social and cultural activities offered by the Museu da Vida, the cultural branch of Fiocruz, and transform this environment into a “park campus” open to the public. Beyond depicting the history and memory of the institution and the scenario of science and health in Brazil, this study attempted to understand the NAHM as an inseparable part of a territory, and as such included a qualitative and quantitative survey directed at staff of the institution and local residents to consolidate a diagnostic of social participation within the scope of this plan. These authors found that mechanisms are still required to reach and involve these groups within the set of projects and initiatives which are being outlined in order to effectively implement not only the directive for social participation which is present in the reference documentation for the NAHM Requalification Plan, but especially to consolidate the social vocation of this cultural heritage and collaborate to promote sustainable development of the territory.

The essay by Renata Reis entitled “Remember, recognize, revere: spaces for the memory of the technical staff at Fiocruz” presents the outcome of an educational heritage project on the historical and affective living spaces in the Manguinhos campus related to memory and to the many stories of the laboratory assistants who worked at the Instituto Oswaldo Cruz (IOC) during its first 30 years. The purpose of this initiative was to establish a memory space for the institution’s technical staff; it was designed to promote dialog between the past and the present in the history of Fiocruz, integrating virtual and in-person environments. The goal was for the entire Fiocruz community and the general public to explore aspects of the work relationships at the old IOC and the many stories of its staff who worked side by side with the scientists and also helped to build Brazilian science and public health. The project is linked to the Manguinhos Historical Architectural Complex Requalification Plan and received support from the call for projects related to the Institutional Memory of Fiocruz in 2020.

The “Interview” section features an interview with the artist, teacher, and author of Brazilian folk verses known as *cordel* Leonardo de Souza Melo (Leo Salo); he is also the founder of the Experimentalismo Brabo project, a provocative art collective that emerged in the Manguinhos Favela Complex (Rio de Janeiro-RJ), and spoke with Elis Regina Barbosa Angelo about the process of interaction that took place within this territory. The conversation highlights the process of development that resulted in the collection of stories of the Manguinhos Favela Complex told through popular poetry and recorded in *cordel* booklets.

Finally, the “Images” section records the experience of workshops on heritage education in the Manguinhos community with the text “Fighting against erasure: a community archive of photos of the São Daniel Profeta Church in Manguinhos”, by Inês El-Jaick Andrade and Éric Alves Gallo. The article focuses on a selection of historic photographs from an album related to that faith community, presenting the social context in which these images circulated. It discusses the interpretation of that constructed heritage and the building of recognition and value attributed to the tangible and intangible ties that connect the church and its community within the territory of Manguinhos.

Understanding health in its broadest sense allows it to be approached in an increasingly far-reaching and inclusive manner. Memory, identity, territory, and culture are important components of this vision, to which the texts gathered here in this supplement are intended to contribute. We hope you enjoy it.
